# Novel nomogram for predicting the progression of osteoarthritis based on 3D-MRI bone shape: data from the FNIH OA biomarkers consortium

**DOI:** 10.1186/s12891-021-04620-y

**Published:** 2021-09-12

**Authors:** Yingwei Sun, Chunbo Deng, Zhan Zhang, Xun Ma, Fenghua Zhou, Xueyong Liu

**Affiliations:** 1 Department of Radiology, Shengjing Hospital of China Medical University, Shenyang, Liaoning Province China; 2 Department of Radiology, Affiliated Hospital of Liaoning University of Traditional Chinese Medicine, Shenyang, Liaoning Province China; 3 Department of Orthopedics, Shengjing Hospital of China Medical University, Shenyang, Liaoning Province China; 4 Department of Orthopedics, Central Hospital of Shenyang Medical College, Shenyang, Liaoning Province China; 5 Department of Rehabilitation, Shengjing Hospital of China Medical University, No.16, Puhe Street, Shenyang North New Area, Liaoning Province 110134 Shenyang, China

**Keywords:** Bone shape, Osteoarthritis, Nomogram

## Abstract

**Background:**

Osteoarthritis(OA) is a major source of pain, disability, and socioeconomic cost in worldwide. However, there is no effective means for the early diagnosis of OA, nor can it accurately predict the progress of OA. To develop and validate a novel nomogram to predict the radiographic progression of mild to moderate OA based on three-dimensional(3D)-MRI bone shape and bone shape change during 24 months.

**Method:**

Analysis of publicly available data from the Foundation for the National Institutes of Health (FNIH) OA Biomarkers Consortium. Radiographic progression was defined as minimum radiographic narrowing of the medial tibiofemoral joint space of ≥ 0.7 mm from baseline at 24, 36, or 48 months. There were 297 knees with radiographic progression and 303 without. The bone shapes of the tibia, femur, and patella were evaluated by 3D-MRI at the baseline and at 24 months. Two nomograms were separately established by multivariate logistic regression analysis using clinical risk factors, bone shape at baseline (nomogram 0), or bone shape change at 24 months (nomogram Δ24). The discrimination, calibration, and usefulness were selected to evaluate the nomograms.

**Results:**

There were significant differences between groups in baseline Kellgren-Lawrence (KL) grade, gender, age, and tibia, femur, and patella shape. The areas under the curve (AUC) of nomogram 0 and nomogram Δ24 were 0.66 and 0.75 (*p* < 0.05), with accuracy of 0.62 and 0.69, respectively. Both nomograms had good calibration. The decision curve analysis ( DCA) showed that nomogram Δ24 had greater clinical usefulness than nomogram 0 when the risk threshold ranged from 0.04 to 0.86.

**Conclusions:**

Nomograms based on 3D-MRI bone shape change were useful for predicting the radiographic progression of mild to moderate OA.

## Background

Osteoarthritis (OA) is one of the most common musculoskeletal disorders in middle-aged and elderly patients. There are currently no successful disease modifying therapies, and OA has been a major cause of disability in the elderly [1–3]. Within the joint, the subchondral bone and articular cartilage are regarded as a whole, called the osteochondral unit. The osteochondral unit has obvious changes in composition, functional properties, structure, and cell activities during the development of OA [4, 5]. In particular, the interaction (crosstalk) between cartilage and subchondral bone, the subchondral microenvironment, has a critical role in the progression of osteoarthritis [6, 7]. Therefore, subchondral bone is expected to serve as an imaging biomarker and a therapeutic target for OA [8].

Plain radiography is the standard imaging technique for diagnosing OA. However, plain radiography does not provide information about the condition of articular cartilage, ligaments, and other tissues. Furthermore, there is a time lag in reflecting changes in the bone. Plain radiography can only detect joint space narrowing and osteophytes. It is not suitable for the early diagnosis of OA, nor can it accurately predict the progression of OA [9]. MRI is a 3D imaging technique that can directly visualize and analyze the whole knee structure, including bone shape. Neogi et al. used an active appearance model to measure knee bone shape parameters on MRI and found that bone shape change began at least a year or more before the onset of radiographic OA (ROA). When the KL grade remains unchanged, abnormal changes in MRI bone shape can predict the occurrence of OA. Three dimensional bone shape imaging can provide more accurate or better visualization than plain radiography, and the accuracy of analysis will not be affected by problems such as projection, rotation, and resolution [10]. Hunter et al. found that changes in the subchondral bone area and 3D bone shape at 24 months were associated with progression of OA [11]. Because a single image biomarker is not enough to predict the progress of OA, they also tried to screen for an optimal combination of imaging and biochemical biomarkers to predict the progression of OA [12].

Compared with other disciplines including oncology, neuroscience, and genomics, the use of advanced analytical techniques in the field of OA is relatively rare [13]. A nomogram is a pictorial representation of a complex mathematical formula that uses certain demographic, clinical, or treatment variables to graphically depict a statistical prognostic model. Nomograms are now widely used in oncology and other disciplines to assist with prediction of disease prognosis, and clinical decision making is refined as humanized digital interfaces help to improve accuracy and make the prognosis easier to understand [14]. Nonetheless, our review of the literature confirms that the nomogram is rarely used to predict the progression of OA. In this study, we aimed to construct nomograms based on 3D-MRI bone shape which was early researched by Professor Hunter to predict the radiographic progression of knee OA and enhance predictive capability for clinicians and researchers.

## Materials and methods

### Study participants

The data used in this study are from datasets of the FNIH biomarkers consortium project within the Osteoarthritis Initiative (OAI). The FNIH OA biomarker project included 600 participants with KL grade 1–3 knees. The 600 participants were divided into 4 different outcome groups at the end of follow-up: (1) participants with both radiographic and pain progression (*n* = 194); (2) participants with only radiographic progression (*n* = 103); (3)participants with only pain progression (*n* = 103); and (4) participants with neither radiographic nor pain progression (*n* = 200) [11]. In this study, we examined radiological progress as the outcome event and the radiographic progression group included 297 individuals who had narrowing of the minimum medial tibiofemoral joint space width (min JSW) of at least 0.7 mm from baseline at 24, 36, or 48 months. The control group included 303 individuals who showed no such progression during the observation period [15].

### Magnetic resonance imaging parameters and 3D-MRI subchondral bone area measurement

MRI acquisition (3T) was performed at four OAI clinical sites using Siemens Trio 3T MRI systems (Siemens Healthcare, Erlangen, Germany). The pulse sequence protocol consisted of 2D coronal intermediate-weighted (IW) turbo spin-echo with 3D sagittal dual-echo at steady-state (DESS) and coronal and axial multiplanar reformations plus sagittal IW fat-saturated turbo spin echo (TSE) sequences [16]. The measurements of 3D-bone shape were published previously [11]. In brief, the 3D-MRI bone shape was reconstructed by active appearance models (AAMs). AAMs were constructed from training sets of 96 examples obtained using the DESS-we sequence for the femur, tibia, and patella [17]. First, the whole bone was built using the piecewise affine registration method, which takes the whole of the MRI image around the knee for each member and generates a statistical model for the whole volume. During model construction, each member was populated with a dense network of control points throughout the volume [18]. Then, AAMs were applied to automatically segment the anatomical regions of bone shape. The definition was modified to include the bone around the cartilage plate (peripheral osteophytes) [11]. Finally, 3D vectors were used to quantitatively analyze the 3D-MRI bone shape. The changes in the position of the femur, tibia, and patella were assessed by 3D shape vectors, where the OA vector was the line that is established by taking the principal components of the mean non-OA shape and the mean OA shape. Each bone shape was projected orthogonally onto the vector and distances along the vector were normalized (z-score). The mean OA and non-OA bone shapes are expressed as -1 and + 1, respectively [11].

In this study, we analyzed the shape of the entire femur, the entire tibia, and the entire patella at baseline and the corresponding changes in subchondral bone area from baseline to 24 months.

### Statistical analysis

Data analysis was conducted using IBM SPSS, version 22.0, Empower (R) (http://www.empowerstats.com, X & Y Solutions Inc., Boston MA) and R (R 3.6.1 http://www.Rproject.org). Continuous variables were presented as means ± standard deviation (SD). Data of normal distribution were tested using independent-samples t-test and data with non-normal distribution were analyzed by Mann-Whitney U test. Categorical variables were expressed as number (percent) (N (%)) and analyzed with chi-square and fisher exact test. All tests were two-sided, and the significance level was set as *p* < 0.05. Backward stepwise multivariate logistic regression analysis was used to establish the nomograms by screening for clinical risk factors, subchondral bone area at baseline (nomogram 0), and subchondral bone area change at 24 months (nomogram Δ24). We used the likelihood ratio test (LRT) of the Akaike information criterion (AIC) as the stop criterion of the stepwise reverse logistic regression analysis. A variance inflation factor (VIF) was leveraged to analyze the co-linearity of various factors in the logistic regression analysis. VIF > 10 was considered indicative of multi-collinearity.

The clinical risk factors included age, gender, body mass index (BMI), race, minJSW, KL grade, and Western Ontario and McMaster Universities Arthritis Index (WOMAC) pain (WOMKP) and function score (WOMADL). In the OAI, the categories for race are named 0 to 3, with 0 representing Non-white/Other; 1, White/Caucasian; 2, Black/ African American; and 3, Asian. We generated nomograms on the basis of the multivariate analysis. Area under the curve (AUC) and accuracy based on the receiver operating characteristic (ROC) curve were measured to test the discrimination of the nomograms, and the AUCs for nomogram 0 and nomogram Δ24 were compared using DeLong’s test. Furthermore, the calibration curve was used to evaluate the calibration of the nomogram [19]. The optimal calibration was defined as the coincidence of the calibration curve and the diagonal. Meanwhile, a Hosmer-Lemeshow (HL) test was performed (*p* > 0.05, supporting the calibration). The internal validation was completed by bootstrapping of 1,000 repeated samplings to reduce the bias of excessive fitting. Decision curve analysis (DCA) was performed to determine the clinical validity of the nomogram by measuring the net benefits at different threshold probabilities. The net benefit was calculated by subtracting the proportion of all patients who are false positive from the proportion who are true positive, weighting by the relative harm of forgoing treatment compared with the negative consequences of an unnecessary treatment. The x axis of the DCA is the threshold of the predicted probability using the combined nomogram to classify knees with and without radiographic progression. The y axis shows the clinical decision net benefit for subjects based on the classification result in this threshold [20].

## Results

### Baseline data of the participants

A detailed comparison of the case and control groups is shown in Table 1. There were significant differences at baseline in age, gender, KL grade, and bone shapes of the tibia, femur, and patella (*p* < 0.05). The groups had similar (*p* > 0.05) BMI, WOMKP, WOMADL, race and minJSW.

**Table 1:** Baseline characteristics of the study groups

	Control cohort (*n* = 303)	Case cohort (*n* = 297)	*p*-value
BMI, kg/m^2^	30.70 ± 4.85	30.73 ± 4.72	0.998
minJSW	3.88 ± 1.01	3.78 ± 1.32	0.16
Age, yr	60.69 ± 9.04	62.42 ± 8.65	0.017
WOMADL	8.32 ± 10.85	9.00 ± 10.43	0.226
WOMKP	11.82 ± 15.30	12.36 ± 15.95	0.513
KL grade, n (%)			0.002
1	37 (12.21 %)	38 (12.79 %)	
2	175 (57.76 %)	131 (44.11 %)	
3	91 (30.03 %)	128 (43.10 %)	
Female sex, n (%)	197 (65.02 %)	156 (52.53 %)	0.002
Race, n (%)			0.691
0: Other Non-white	6 (1.48 %)	5 (2.58 %)	
1: White	320 (78.82 %)	155 (79.90 %)	
2: White or Caucasian	77 (18.97 %)	32 (16.49 %)	
3: Aian	3 (0.74 %)	2 (1.03 %)	
Baseline bone shape			
Femur OA Vector	0.50 ± 2.60	0.01 ± 2.58	< 0.001
Tibia OA Vector	-0.03 ± 1.15	-0.34 ± 1.25	0.002
Patella OA Vector	0.01 ± 1.57	-0.56 ± 1.74	< 0.001

Femur OAVector the femur bone shape, Tibia OAVector the tibia bone shape,

Patella OAVector the patella boneshape

### Establishment of the prediction nomograms

**Fig. 1:**
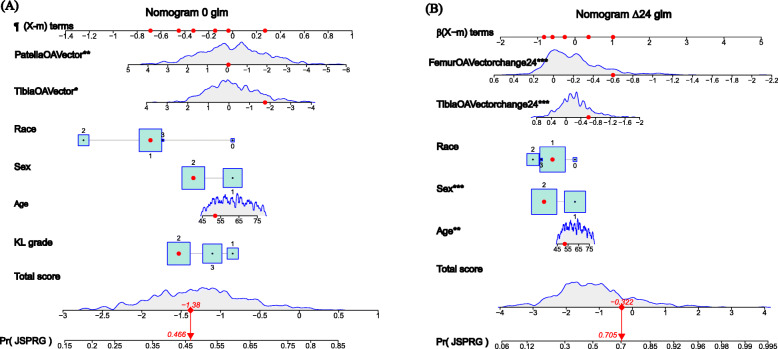
Construction of 3D-MRI nomograms. The distribution and total point number of predictive variables overlap along the nomogram scales. The box plot shows the categorical variables (e.g., KL grade, race, and gender), with the box size indicating percentage. The density plot shows the distribution of continuous variables (e.g., 3D bone shape of tibia and patella at the baseline, 3D bone shape changes of tibia and femur at 24 months of follow-up, age, and total score). The definite value of each red point corresponds to the scale of the variable’s axis (β(χ-m) term). The observation values overlap at the total score axis. A straight line is drawn through this point and downward extending to the risk axis, and the point of intersection with the risk axis represents the occurrence probability of the radiographic progression of osteoarthritis. **A** Nomogram for 3D bone shape at the baseline. **B** Nomogram for 3D bone shape change at 24 months

For nomogram 0, the backward stepwise multivariate logistic regression analysis identified 3D tibial and patellar bone shape at baseline, race, KL grade, gender, and age as risk factors. Tibial and femoral bone shape change at 24 months, race, gender, and age were included as risk factors in nomogram Δ24 (Table 2; Fig. 1). The advantage of the nomogram is that it transforms the complex regression equation into a simple and visual graph, which makes the results of the prediction nomogram more readable and has higher use value. According to the degree of influence of various covariates in the nomogram on the outcomes (the size of the regression coefficient), each value level of each covariate was assigned a score. Regardless of statistical significance, the cumulative total score of various covariates corresponds to the prediction probability of the subjects. Figure 1a shows a multivariate-nomogram as an example. The box plot shows the categorical variables, with the box size indicating percentage. The density plot shows the distribution of the continuous variables. The red point on each variable corresponds to the scale of the variable axis (β(χ-m) term). When the scores of individual predictors are added, a total score of -1.38 corresponds to a probability of progression of ROA of 46.6 %. Figure 1b shows that for the nomogram Δ24, when scores for individual variables are added, a total score of -0.322 indicates a probability of progression of ROA as high as 70.5 %.

**Table 2:** Multivariate binary logistic regression analysis for predictors of osteoarthritis progression

Time	Intercept and variable	β	OR	95% CI.low	95% CI.upp	*p*-value
**Baseline**	Intercept	0.1749				0.856
	KL2	-0.4584	0.6323	0.3705	1.0790	0.093
	KL3	-0.1662	0.8468	0.4711	1.5221	0.578
	Age, year	0.0158	1.0159	0.9965	1.0356	0.108
	Sex, female	-0.3020	0.7393	0.5157	1.0601	0.100
	Race 0, Other Non-white	Reference	Reference	Reference	Reference	Reference
	Race 1, White or Caucasian	-0.6816	0.5058	0.1276	2.0050	0.332
	Race 2, Black or African American	-1.2803	0.2779	0.0671	1.1507	0.077
	Race 3, Asian	-0.5724	0.5642	0.0539	5.9064	0.633
	Tibia OA Vector	-0.1646	0.8483	0.7268	0.9901	0.037
	Patella OA Vector	-0.1748	0.8380	0.749	0.9375	0.002
**24 m**	Intercept	-1.4507				0.143
	Age, year	0.0295	1.0299	1.0093	1.0509	0.004
	Sex, female	-0.8186	0.4422	0.3039	0.6434	0.000
	Race 0, Other Non-white	Reference	Reference	Reference	Reference	Reference
	Race 1, White or Caucasian	-0.6009	0.5483	0.1308	2.2989	0.411
	Race 2, Black or African American	-1.1677	0.3111	0.0702	1.3784	0.124
	Race 3, Asian	-0.9037	0.4039	0.039	4.1849	0.447
	Femur OA Vector change24	-2.6689	0.0693	0.0315	0.1528	0.000
	Tibia OA Vector change24	-1.0035	0.3666	0.2216	0.6066	0.0001

### Validation of the nomograms

**Fig. 2:**
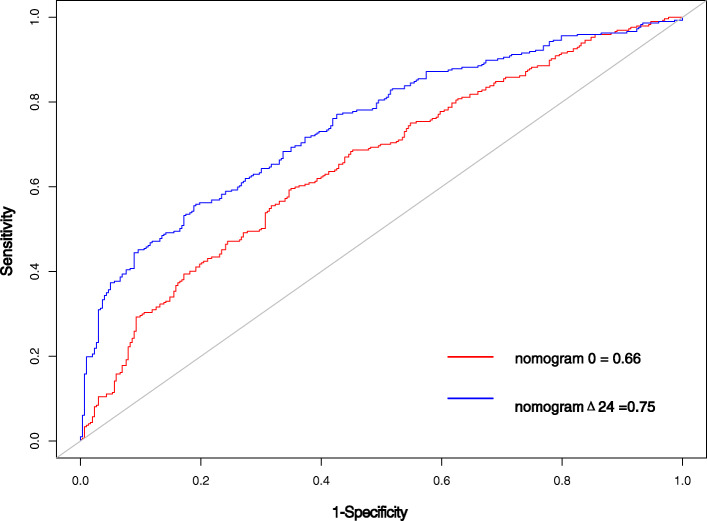
Receiver operator characteristic curves for nomogram 0 and nomogram Δ24. The AUC for nomogram Δ24 was significantly higher (*p* < 0.05)

**Fig. 3:**
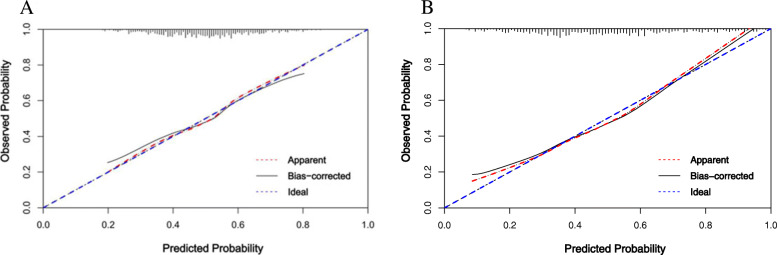
Calibration curves of models. **A** Nomogram 0 and **B** Nomogram Δ24 showing predicted progression (X axis) vs. actual progression (Y axis). The dashed diagonal line represents the ideal prediction of the ideal model and the solid line represents the performance of the nomogram; the areas of closer approach indicate better prediction

**Fig. 4:**
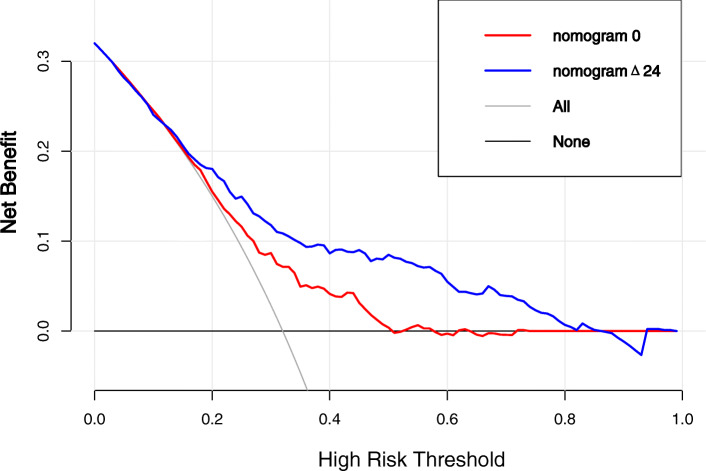
Decision curve analysis for nomogram Δ24 compared nomogram 0 predicted the radiological progression of OA. The Y axis shows the net benefit. The x axis of DCA is the threshold of the predicted probability using the nomogram to classify subjects with radiographic progression and subjects without radiographic progression. The gray line represents the hypothesis that all subjects had OA radiological progression; the black line represents the hypothesis that none of the subjects had any radiological progression of OA. The net benefit of using the nomogram Δ24 (blue line) for clinical decision-making exceeds that of using nomogram 0 (red line) across the range of reasonable risk thresholds

The VIF of all predictors in both nomograms were < 5 in every instance, indicating that there is no collinearity between variables. The AUC of nomogram Δ24 (0.75, 95 % CI [confidence interval]: 0.71 to 0.79) was significantly higher than the AUC of nomogram 0 (0.66, 95 % CI 0.61 to 0.71)(Fig. 2). The accuracy of nomogram 0 and nomogram Δ24 were 0.62 and 0.69 respectively. The Hosmer-Lemeshow test shows that the non-significant p values of nomogram 0 and nomogram Δ24 are 0.21 and 0.34, respectively, and the calibration curve (Fig. 3) shows that the prediction results of nomograms were consistent with the actual results, indicating good calibration in predicting the progression of ROA. The x axis of the DCA is the threshold of the predicted probability using the combined nomogram to classify knees with and without radiographic progression. The y axis shows the clinical decision net benefit for subjects based on the classification result in this threshold. The gray line represents the hypothesis that all subjects had progression of ROA (progress all); the black line represents the hypothesis that none of the subjects had any progression of ROA (progress-none). The DCA showed that nomogram 0 offered a net benefit over the progress all or progress-none strategy at a threshold range of roughly 12 to 57 %, and nomogram Δ24 offered a net benefit over the progress all or progress-none strategy at a threshold range of roughly 4 to 86 %. The benefit of nomogram Δ24 is higher than nomogram 0 at a threshold range roughly 4 to 86 % (Fig. 4).

## Discussion

We developed and validated nomograms to predict the radiological progression of OA based on 3D-MRI bone shape and clinical risk factors. The nomogram at baseline (nomogram 0) incorporates tibial bone shape and patellar bone shape, race, KL grade, gender, and age. The nomogram at 24 months (nomogram Δ24) incorporates the change of tibial and femoral bone shape, race, gender, and age. Our study demonstrates that the nomograms could predict the progression of ROA.

Although there are some studies on the correlation between bone shape and the progression of OA, we think our study has verified the previous work with a novel method that has been widely used in oncology and other disciplines. The bone structure changes correspondingly to adapt to changes in mechanical load [21]. The deterioration of the 3D trabecular bone structure in femoral condyles was a more sensitive indicator of early OA [22], and changes in the shape of the femoral surface have been used to predict the need for knee replacement [23]. Hunter et al. found correlation between changes in shape over 24 months and a combination of radiographic and pain progression in OA [11]. Osteophytes may promote the increase of bone surface area, because previous studies have suggested that subtle alterations in joint shape at knee may be involved in the pathogenesis of OA, so Hunter also included osteophytes as the boundary of bone morphology [11]. In our study, the stepwise reverse logistic regression analysis of a nomogram was based on the AIC principle, not on *p* value. The prediction probability is determined by the effect estimates of the variable, regardless of statistical significance, and it is influenced by the presence of other covariates. Therefore, both nomogram 0 and nomogram Δ24 contain variables with *p* values > 0.05, which are different from traditional regression models. Nomogram 0 incorporates patellar bone shape, which was rarely used to predict decreased medial femorotibial compartment joint space width. In a recent study, the patella vector at the baseline was one of the best variables among the combined imaging and biochemical biomarkers for predicting OA in the FNIH study [12]. This shows that the nomogram model may contain the most predictive variables. In addition, by comparing the discrimination, calibration, and usefulness of the two nomograms, we found that nomogram Δ24 including the changes of bone shape had better predictive performance. Therefore, it can be proven that the changes in bone shape are related to the progression of OA.

In our study, five independent variables of nomogram Δ24 were confirmed as predictors of the progression of ROA. The changes of bone shape have the largest weight in nomogram Δ24. Narrowing of the knee joint space is influenced by many factors including thinning articular cartilage, bone marrow lesions (BMLs), meniscus extrusion, etc. [24–26]. All of the above factors are related to bone shape. Eckstein et al. confirmed thinning of the medial tibiofemoral articular cartilage during the progression of OA [24], while the thickness of the medial tibial subchondral plate increased significantly [27]. Zhong et al. measured the bone shape change after anterior cruciate ligament (ACL) reconstruction (ACLR) from baseline to 36 months post-surgery and examined the relationship between the change in bone shape from baseline to 6 months after surgery and the changes in the articular cartilage matrix and the function and symptoms of patients after 36 months [28]. The group found that early bone shape change (within 6 months) in patients with partial ACL injury was related to both prognosis and the condition of the cartilage matrix after 36 months. The authors proposed that the change of bone shape could be an imaging biomarker for identifying patients at high-risk of traumatic osteoarthritis [28]. Snoeker et al. found that when ACL injury is combined with meniscus injury, the bone area corresponding to the meniscus increases by about 1 %, which indicates that there is a potentially important relationship between the integrity of the meniscus, early meniscus injury, and changes in bone surface area after ACL injury [29]. We can speculate that meniscal damage accelerates the changes in bone shape. BMLs have been associated with pain in OA [25]. BMLs are also positively correlated to bone shape changes, and bone shape change has shown a higher correlation with OA progression than BMLs [30, 31]. Unlike BMLs, which are not seen in every case of OA, we believe that shape changes occur in all cases of OA, and OA patients with abnormal bone shape can be regarded as a special high-risk group.

In addition, gender, age, and race in nomograms are used as predictors of OA progression, and their relationship to the progression of OA has been reported in previous studies [32, 33]. The independent variables (gender, age, and race) in the nomogram are also related to the change of bone shape. The baseline 3D knee shape varies among races [34], and we also found that race was an important factor in the progression of OA in this study. Barton et al. found that there was a variation between genders in the bone shape of the knee and observation of the bone shape of the knee from baseline to 48 months found that although the shapes of the distal femur and proximal tibia did not change much over time, both gender and the baseline KL grade were related to the change trajectory of the bone shape [35, 36]. In another study by Barton’s group, a regression analysis that did not include specific bone shapes showed that gender had a direct impact of > 1 on the risk of ROA of the knee, suggesting that women were at higher risk of knee ROA. However, a regression analysis that included gender and bone shape showed an effect of gender on the ROA risk of < 1 for the knee, indicating that certain specific bone shapes could protect women from a higher ROA risk. The shapes of the distal femur and proximal tibia partially and inconsistently mediated the relationship between gender and incident knee OA. Women had a higher risk of incident ROA, and specific bone shapes were associated with modest protection from an even greater risk [37], and the mechanism is not clear. In our two nomograms, the contribution of female gender to predicting the progression of OA was lower than that of male gender, which contradicts the traditional view that women are more likely to develop OA. The 3D-MRI measurements of the surface areas of the knee joints indicated that the surface area of the tibial subchondral bone changed adaptively to the abnormal load at a poor force line of the knee joint. Longitudinal observation shows that such an adaptive process might occur more frequently in elderly individuals [38], and the relationship between age, gender, and the change of bone shape, as well as their mechanisms in OA progression, invites further in-depth analysis.

There are certain limitations to this study. First, the changes of the bone shape in this study are changes in the entire patella, distal femur, and proximal tibia, but the change in local areas might be more sensitive. Calculating the changes of bone shape in different areas of the knee joint can improve the discrimination of the model [39]. That is, selection of the appropriate regions of interest (ROIs) determines the predictive ability of the model. It is necessary to segment the bone shape of knee joint and select more accurate ROIs. Second, many important covariates including age, KL, gender, and race, that may cause changes in bone shape, were adjusted in this study. However, the history of injury, surgery, and family genetic history of OA are not considered as covariates. Meanwhile, any load change associated with kinematic changes may lead to bone shape changes [40]. Other genetic factors can also influence bone shape in the knee. Experimental studies have also shown that a GDF5-UQCC1 mutant (rs6060369) can induce a shape change in the knee joints and precedes OA in mice [41]. The correct choice of clinical covariates can also improve the predictive performance of the model. Third, the cohort study design itself has certain limitations. Bone shape change was defined as the change from the baseline to 24 months of follow-up, while the narrowing of the joint space was assessed at baseline and at 24 to 48 months. Therefore, instead of having an absolute predictive relationship, there were some overlapping periods in the study.

Finally, this study is a multi-center study with internal validation. Further external validation is needed to verify the nomograms.

## Conclusions

In summary, nomograms based on 3D-MRI bone shape have good predictive value for OA progression, and the bone shape change at 24 months has better predictive value than bone shape at baseline, which highlights the close relationship between bone shape change and OA progression.

## Data Availability

All data are available from the OAI of the FNIH (https://data-archive.nimh.nih.gov/oai/).

## Abbreviations

3D: Three-dimensional
OAI: Osteoarthritis initiative
FNIH: The Foundation for the National Institutes of Health
KL: Kellgren-Lawrence
AUC: The areas under curve
minJSW: Minimum joint space width
WOMKP: WOMAC pain score
WOMADL: WOMAC function score
LRT: Likelihood ratio test
AIC: Akaike information criterion
VIF: Variance inflation factor
DCA: The decision curve analysis
BMLs: Bone marrow lesions
ACLR: Anterior cruciate ligament reconstruction
